# Curcumin (a constituent of turmeric): New treatment option against COVID‐19

**DOI:** 10.1002/fsn3.1858

**Published:** 2020-09-06

**Authors:** Fatemeh Babaei, Marjan Nassiri‐Asl, Hossein Hosseinzadeh

**Affiliations:** ^1^ Department of Clinical Biochemistry School of Medicine, Student Research Committee Shahid Beheshti University of Medical Sciences Tehran Iran; ^2^ Department of Pharmacology and Neurobiology Research Center School of Medicine Shahid Beheshti University of Medical Sciences Tehran Iran; ^3^ Department of Pharmacodynamics and Toxicology School of Pharmacy Mashhad University of Medical Sciences Mashhad Iran; ^4^ Pharmaceutical Research Center Pharmaceutical Technology Institute Mashhad University of Medical Sciences Mashhad Iran

**Keywords:** antiapoptotic, antifatigue, antifibrotic, anti‐inflammatory, antiviral, Coronavirus‐19, curcumin

## Abstract

In late December 2019, the outbreak of respiratory illness emerged in Wuhan, China, and spreads worldwide. World Health Organization (WHO) named this disease severe acute respiratory syndrome coronavirus 2 (SARS‐CoV‐2) caused by a new member of beta coronaviruses. Several medications are prescribed to patients, and some clinical trials are underway. Scientists are trying to find a specific drug against this virus. In this review, we summarize the pathogenesis, clinical features, and current treatments of coronavirus disease 2019 (COVID‐19). Then, we describe the possible therapeutic effects of curcumin and its molecular mechanism against coronavirus‐19. Curcumin, as an active constituent of *Curcuma longa* (turmeric), has been studied in several experimental and clinical trial studies. Curcumin has some useful clinical effects such as antiviral, antinociceptive, anti‐inflammatory, antipyretic, and antifatigue effects that could be effective to manage the symptoms of the infected patient with COVID‐19. It has several molecular mechanisms including antioxidant, antiapoptotic, and antifibrotic properties with inhibitory effects on Toll‐like receptors, NF‐κB, inflammatory cytokines and chemokines, and bradykinin. Scientific evidence suggests that curcumin could have a potential role to treat COVID‐19. Thus, the use of curcumin in the clinical trial, as a new treatment option, should be considered.

## Graphical abstract

Curcumin has antinociceptive, antipyretic, antifatigue, and antiemetic effects. Curcumin could alleviate symptoms of the infected patient with COVID‐19. Curcumin could reduce inflammatory cytokines via inhibiting TLR/MAPK/NF‐κB signaling. Curcumin might attenuate ARDS and pulmonary fibrosis due to anti‐inflammatory properties.

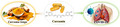

## 1 INTRODUCTION

In late December 2019, the outbreak of respiratory illness was reported in Wuhan, China. After a while, the cause of this unknown pneumonia was recognized as a novel coronavirus named severe acute respiratory syndrome coronavirus 2 (SARS‐CoV‐2) by World Health Organization (WHO) (Zhu et al., 2020; He, Deng, & Li, 2020; Huang et al., 2020).

Coronaviruses (CoVs) are enveloped positive‐stranded RNA viruses that cause respiratory, enteric, hepatic, and neurological diseases in humans and animals (Zumla, Chan, Azhar, Hui, & Yuen, 2016). Some human CoVs, such as HCoV‐OC43, HCoV‐HKU1, HCoV‐229E, and HCoV‐NL63, create mild respiratory illness, but some others including severe acute respiratory syndrome CoV (SARS‐CoV) and Middle East respiratory syndrome CoV (MERS‐CoV) cause severe diseases (Li, Bai, & Hashikawa, 2020). It is identified that COVID‐19 transmitted among humans by respiratory droplets and close contact (Chan et al., 2020). A recent research project revealed that sequence homology between SARS‐CoV‐2 and SARS‐CoV was 79.5% (Wu et al., 2020) and SARS‐CoV‐2 belongs to the same beta coronavirus (β CoV) clade as MERS‐CoV and SARS‐CoV (Yu, Du, Ojcius, Pan, & Jiang, 2020). Moreover, it has been identified that SARS‐CoV‐2 had high homology with bat coronaviruses and likely derived from bats (Zhou et al., 2020), but the intermediate hosts of SARS‐CoV‐2 have not been determined yet. The recent finding reveals that COVID‐19 has similar pathogenesis with SARS‐CoV or MERS‐CoV (Song et al., 2019) and uses the same receptor as SARS‐CoV for entrance to human host cells (Lu et al., 2020; Wan, Shang, Graham, Baric, & Li, 2020).

## 2 METHODS

The most important articles about COVID‐19 (from starting disease up to now) and curcumin were selected. We considered all articles of curcumin—human and animal studies—that could be effective to treat or rescue COVID‐19‐infected patients. PubMed and Web of Science were used as databases. As the importance of the subject, some selected papers were in the press. The keywords used for the search were as follows: coronavirus‐19, COVID‐19, SARS‐CoV‐2, curcumin, *Curcuma longa*, turmeric, curcumin and antiviral, curcumin and anti‐inflammatory, curcumin and antipyretic, curcumin and lung, curcumin and acute lung injury, curcumin and fatigue, curcumin and antioxidant, curcumin and ARDS, curcumin and bradykinin, curcumin and fibrosis, curcumin and Interleukin‐6 (IL‐6), curcumin and tumor necrosis factor‐alpha (TNF‐α), curcumin and NF‐κB, curcumin and Toll‐like receptors (TLRs), curcumin and antiapoptotic.

## 3 PATHOGENESIS

The SARS‐CoV‐2 is an enveloped nonsegmented positive‐sense RNA virus. Two‐thirds of viral RNA is located in the first open reading frames that encode 16 nonstructural proteins, whereas the remaining part of the genome encodes four essential structural proteins including spike (S) glycoprotein, envelope (E) protein, matrix (M) protein, and nucleocapsid (N) protein (Cui, Li, & Shi, 2019).

S protein contributes to virus pathogenesis through binding to cell surface receptor, angiotensin‐converting enzyme 2 (ACE2), and the entrance of the virus into the host cell (Zhou et al., 2020). The possible mechanism and molecules involved in membrane invagination during virus endocytosis are still unknown. S protein is divided into the S1 domain that is responsible for receptor binding, and S2 domain that mediates cell membrane fusion (He et al., 2004).

Recent data showed that the S protein of SARS‐CoV‐2 binds to ACE2 with a higher affinity than SARS‐CoV. For this reason, it spreads rapidly in human populations (Wrapp et al., 2020). The ACE2 expressed on the surface of cells in the lung, arteries, heart, kidney, and intestine (Hamming et al., 2004). Its concentration in the alveolar cells of men was higher than women, which may correlate with a high incidence rate of COVID‐19 among men. Moreover, the expression level of ACE2 in the alveolar cells of Asians was higher than other races, which may lead to high susceptibility to disease and severe outcomes (Sun, Lu, Xu, Sun, & Pan, 2020). The ACE2 is an enzyme that catalyzes vasoactive angiotensin II to vasodilator angiotensin[1–7] (Richards & Raizada, 2018).

On the other hand, the binding of the SARS‐CoV spike protein to ACE2 leads to ACE2 downregulation (Kuba et al., 2005). It is not clear that SARS‐CoV‐2 could downregulate the expression of ACE2 or not as the homology of SARS‐CoV‐2 with SARS‐CoV. ACE2 downregulation resulted in excessive production of angiotensin by the ACE, suggesting lead to pulmonary hypertension, acute lung injury (ALI), and lung fibrosis (Tan, Liao, Zhou, Mei, & Wong, 2018).

Previous studies have shown the protective role of ACE2 against various types of pulmonary illnesses such as acute respiratory distress syndrome (ARDS), chronic obstructive pulmonary disease (COPD), pulmonary hypertension, ALI, and asthma (Jia, 2016). It has been suggested that increasing ACE2 levels such as using angiotensin II receptor blocker medications could be effective to treat COVID‐19 (Gurwitz, 2020). However, another study showed that decreasing ACE2 activity might be protective (Zhang, Penninger, Li, Zhong, & Slutsky, 2020). Thus, these hypotheses might be the basis for more research to clarify new therapeutic options.

### 3.1 Clinical signs and symptoms in patients

The patients mainly were 30–79 years old (Wu & McGoogan, 2020). A few cases were children below 15 years old such as 15 days old in Iran (Kamali Aghdam, Jafari, & Eftekhari, 2020). There were one or more coexisting medical conditions, including hypertension, diabetes, and cardiovascular disease in about half the patients (Chen et al., 2020). These coexisting medical conditions lead to a high mortality rate in COVID‐19 patients (Wu & McGoogan, 2020). There was a spectrum of clinical features ranging from asymptomatic infection to severe respiratory failure. The most prevalent manifestations include fever, fatigue, dry cough, myalgia, dyspnea, and anorexia (Qin et al., 2020; Rodriguez‐Morales et al., 2020). The uncommon symptoms were sputum production, headache, hemoptysis, diarrhea, nausea, and vomiting (Huang et al., 2020; Qian et al., 2020; Rodriguez‐Morales et al., 2020).

### 3.2 Laboratory findings

According to laboratory examination, lymphopenia, hypoalbuminemia, and high levels of C‐reactive protein (CRP), erythrocyte sedimentation rate (ESR), and lactate dehydrogenase (LDH) were the most prevalent results in the patients (Mo et al., 2020; Qin et al., 2020; Rodriguez‐Morales et al., 2020; Talebpour, Hadadi, Oraii, & Ashraf, 2020; Wang, Yang, Li, Wen, & Zhang, 2020).

Patients with severe symptoms had raised levels of coagulation indexes (prothrombin time, activated partial thromboplastin time, and D‐dimer), procalcitonin, IL‐6, and serum ferritin, and multiple organ involvement, such as liver (increased lactate dehydrogenase, alanine aminotransferase, and aspartate aminotransferase levels), kidney (increased blood urea nitrogen and creatinine levels), and heart and muscle (increased creatinine kinase levels) compared with patients with mild symptoms. Also, there was higher neutrophil‐to‐lymphocyte ratio (NLR) and lower percentages of monocytes, eosinophils, and basophils in complete blood count (Qian et al., 2020; Qin et al., 2020; Talebpour et al., 2020; Wan, Xiang, et al., 2020).

The elevated levels of IL‐1B, interleukin‐1 receptor antagonist (IL1RA), IL‐7, IL‐8, IL‐9, IL‐10, basic fibroblast growth factors (FGF), granulocyte colony‐stimulating factor (GCSF), granulocyte‐macrophage colony‐stimulating factor (GM‐CSF), interferon‐gamma (IFNγ), interferon γ‐induced protein 10 kDa (IP‐10), monocyte chemoattractant protein‐1 (MCP1), macrophage inflammatory protein‐1 alpha (MIP1‐α), macrophage inflammatory protein‐1β (MIP‐1β), platelet‐derived growth factor (PDGF), tumor necrosis factor‐alpha (TNF‐α), and vascular endothelial growth factor (VEGF) were observed in serum sample of patients. Plasma levels of IL‐5, IL‐12p70, IL‐15, eotaxin, and RANTES (chemokine (C‐C motif) ligand 5, CCL5) were similar between healthy adults and patients infected with SARS‐COV‐19. Moreover, plasma concentrations of IL2, IL7, IL10, GCSF, IP10, MCP1, MIP1A, and TNF‐α were higher in the intensive care unit (ICU) patients compared with non‐ICU patients. These data suggest that the initial response of the immune system may lead to the production of cytokines and chemokines, which damage normal host organs such as lung and heart. Also, hypersensitive troponin I (hs‐cTnI) was increased in patients with virus‐related cardiac injury (Huang et al., 2020).

### 3.3 Pathology

The pathological features of COVID‐19 were similar to SARS‐CoV and MERS‐CoV infection (van den Brand, Smits, & Haagmans, 2015; Hui & Zumla, 2019; Nassar, Bakhrebah, Meo, Alsuabeyl, & Zaher, 2018). Moreover, moderate microvascular steatosis, mild lobular, and portal activity were observed in the liver biopsy samples. The heart tissue also showed interstitial mononuclear inflammatory infiltrates. The CD4 and CD8 T cells were decreased in flow cytometric analysis of peripheral blood. Also, X‐ray images showed a rapid progression of pneumonia in lung tissues (Xu et al., 2020).

## 4 COVID‐19 AND CURRENT DRUG DEVELOPMENT RESEARCH PROJECTS

Countries use various strategies to treat COVID‐19 patients. Some protocols for the treatment are summarized in Table 1. Some clinical trials try to find an effective drug (Li & De Clercq, 2020). Organ dysfunction, including shock, ARDS, acute cardiac injury, acute kidney injury, liver dysfunction, and secondary inflammation are causes of death among COVID‐19 patients (Chen et al., 2020; Huang et al., 2020; Wan, Xiang, et al., 2020; Wang, Hu, et al., 2020). Current medical therapies are symptomatic treatment or supportive care. There is no definitive treatment yet for this disease. Therefore, finding effective strategies to treat infected patients and protect organs is necessary to decrease the mortality rate.

**Table 1:** Some protocols used for the treatment of COVID‐19

Study details	Treatment protocol	Reference
41 patients, Wuhan, China	Oxygen support, antibiotics (*N*), antiviral (oseltamivir), corticosteroid (methylprednisolone), renal replacement therapy	Huang et al. (2020)
69 patients, Wuhan, China	Oxygen support, antibiotics (*N*), antiviral (*N*), corticosteroid (*N*), antifungal (*N*), arbidol, moxifloxacin, interferon therapy (*N*)	Wang, Yang, et al. (2020)
135 patients, Northeast Chongqing, China	Oxygen support, antibiotics (*N*), antiviral (Kaletra), corticosteroid (*N*), traditional Chinese medicine therapy, renal replacement therapy	Wan, Xiang, et al. (2020)
155 patients, Wuhan, China	Oxygen support, corticosteroid (*N*), expectorant, antiviral: arbidol, lopinavir, ritonavir, interferon inhalation, immune enhancer (thymalfasin, immunoglobulin)	Mo et al. (2020)
One patient Case report, United States	Antipyretic therapy: acetaminophen (650 mg, every 4 hr), and ibuprofen (600 mg, every 6 hr), expectorant: guaifenesin (600 mg), oxygen support, antibiotics (vancomycin and cefepime), antiviral (remdesivir)	Holshue et al. (2020)
99 patients, Wuhan, China	Oxygen support, antibiotics: cephalosporins, quinolones, carbapenems, tigecycline against methicillin‐resistant Staphylococcus aureus, linezolid, Antiviral: oseltamivir (75 mg, every 12 hr), ganciclovir (0.25 g, every 12 hr), and lopinavir and ritonavir (500 mg, twice daily), corticosteroid: methylprednisolone sodium succinate, methylprednisolone, and dexamethasone, antifungal (*N*), renal replacement therapy, immunoglobulin therapy (IV)	Chen et al. (2020)
More than 100 patients Wuhan, Jingzhou, China	Chloroquine phosphate	Gao, Tian, and Yang (2020)
20 patients, Marseille, France	Hydroxychloroquine (600mg, daily)	Gautret et al., (2020)

Note

*N*: not mentioned.

### 4.1 Curcumin as a new option

**Figure 1:**
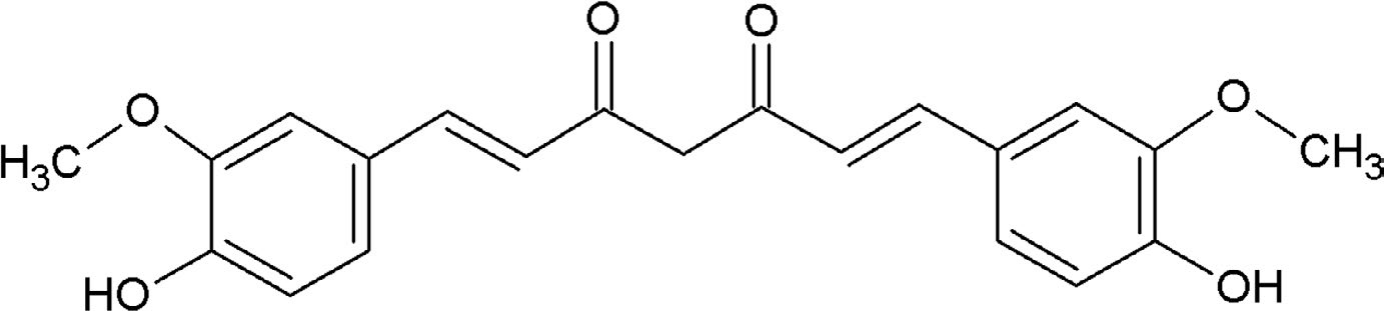
Chemical structure of curcumin

Curcumin, as a potential agent, could be considered to treat COVID‐19. Curcumin, as an active constituent of rhizomes of *C. longa* (turmeric), is a hydrophobic polyphenol (Figure 1) (Akbar et al., 2018; Soleimani, Sahebkar, & Hosseinzadeh, 2018). Curcumin is used as a spice in foods and for different purposes such as cosmetic and pharmaceutical industries in world (Hosseini & Hosseinzadeh, 2018). Curcumin has several pharmacological effects such as antioxidant, anticancer, antibacterial, antiviral, and antidiabetic effects (Fan et al., 2015; Moghadamtousi et al., 2014; Zhu et al., 2017), as well as anti‐inflammatory activity (Cheng, Yang, Hu, Zhu, & Liu, 2018). As the potential role of curcumin to treat many inflammatory disorders, at the first step we will describe all effects of curcumin that may be useful to treat COVID‐19, and then, we explain the possible molecular mechanisms of it.

## 5 THERAPEUTIC EFFECTS OF CURCUMIN AGAINST COVID‐19

### 5.1 Antiviral effects

Curcumin prevented the replication of SARS‐CoV and inhibited 3Cl protease in Vero E6 cells. Also, it significantly has an inhibitory activity against the cytopathogenic effect of SARS‐CoV in Vero E6 cells (Wen et al., 2007). Curcumin was effective against other viruses such as influenza A virus, HIV, enterovirus 71 (EV71), herpes simplex virus (HSV), hepatitis C virus (HCV), and human papillomavirus (HPV) with several mechanisms that made it valuable for antiviral therapies (Moghadamtousi et al., 2014; Praditya et al., 2019; Qin et al., 2014).

Recently, it has shown that the transformation of curcumin into carbon quantum dots could boost antiviral effects of curcumin with different mechanisms against EV71 in vitro and in vivo (Lin et al., 2019). The interesting issue about carbon quantum dots is that it alone was effective against human coronavirus (HCoV) by inhibiting the entry receptor of HCoV‐229E (Łoczechin et al., 2019).

### 5.2 Antiemetic effect

*C. longa* L, as herbal medicine, is used to treat vomiting from ancient times in Asian countries (Liu et al., 2018). Curcumin (20 mg/kg, intragastric, 3 days) improved appetite of rats in chemotherapy induced by fluorouracil (5‐FU) (Yao et al., 2013). It may be effective against vomiting due to COVID‐19.

### 5.3 Reduces myalgia and fatigue

In an animal study, oral administration of curcumin has an antifatigue function and improved physical function in mice (Huang et al., 2015). Administration of curcumin (1,000 mg/d, 30 days) reduced stress and fatigue in the subjects that experiencing occupational stress‐related anxiety and fatigue in a randomized double‐blinded placebo‐controlled study (Pandaran Sudheeran et al., 2016). Curcumin (2.5 g, two times a day) reduced delayed‐onset muscle soreness of healthy men who have a heavy eccentric exercise (Nicol, Rowlands, Fazakerly, & Kellett, 2015). The use of curcumin in myalgic encephalomyelitis/chronic fatigue syndrome as a novel therapeutic option was mentioned (Morris et al., 2019). Curcumin inhibited sepsis‐induced muscle wasting by inhibiting catabolic response in skeletal muscle via blocking NF‐κB (Alamdari, O'Neal, & Hasselgren, 2009). Curcumin (Meriva®, 1 g, 3 months) prevented muscle loss and improved physical performance in healthy elder subjects and delayed the onset of sarcopenia in them (Franceschi et al., 2016). These results suggest that curcumin may be effective to manage myalgia and fatigue symptoms induced by COVID‐19.

### 5.4 Antinociceptive, anti‐inflammatory, and antipyretic effects

The antinociceptive and anti‐inflammatory effects of curcumin in animal and human studies were reviewed by Eke‐Okoro, Raffa, Pergolizzi, Breve, & Taylor, 2018 (Eke‐Okoro et al., 2018). About the important molecular mechanism of these effects, it will discuss later that curcumin could be effective as a novel treatment against COVID‐19. Also, in an animal study, curcumin (100 mg/kg, i.p.) has an antipyretic effect in rats (Haider et al., 2013). It seems that curcumin overcomes the fever of COVID‐19‐infected patients.

### 5.5 Inhibitory effects on cytokines and chemokines

**Figure 2:**
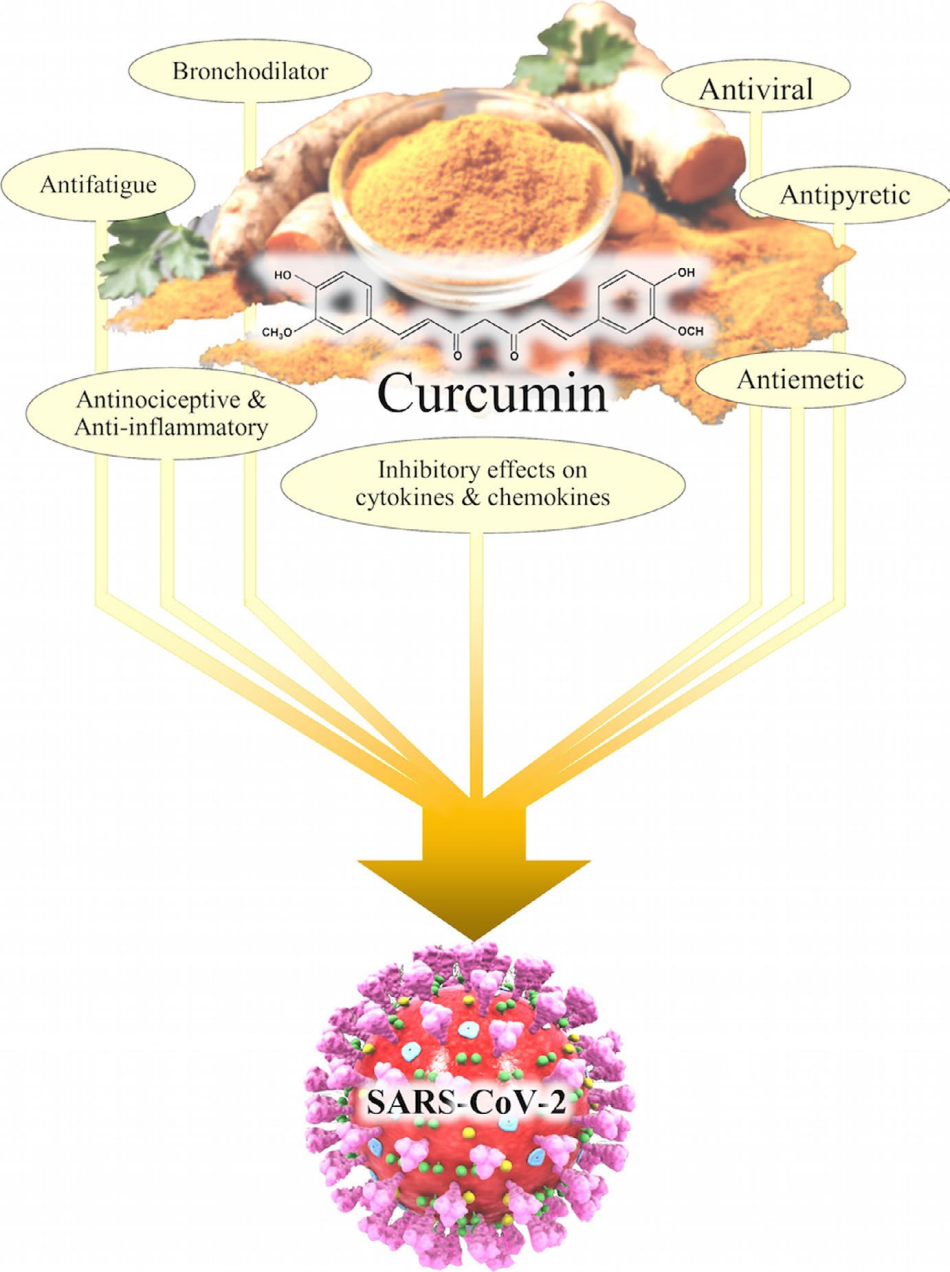
Possible clinical effects of curcumin in the treatment of COVID‐19. SARS‐CoV‐2: severe acute respiratory syndrome coronavirus 2

Two meta‐analyses of randomized controlled trials have shown that curcumin reduced circulating IL‐6 and TNF‐α levels that both are the key inflammatory mediators and increase in inflammatory diseases (Derosa, Maffioli, Simental‐Mendía, Bo, & Sahebkar, 2016; Sahebkar, Cicero, Simental‐Mendía, Aggarwal, & Gupta, 2016). Curcumin also reduced the expression of IL‐1β in M1 macrophages from Behcet's disease patients (Palizgir et al., 2018). Also, curcumin protected human genital mucosal epithelial cells against HIV‐1 replication by inhibiting activation of proinflammatory chemokines such as IL‐8 and RANTES (Ferreira, Nazli, Dizzell, Mueller, & Kaushic, 2015). The summary of the clinical effects of curcumin that may be useful to treat COVID‐19 is illustrated in Figure 2.

**Figure 3:**
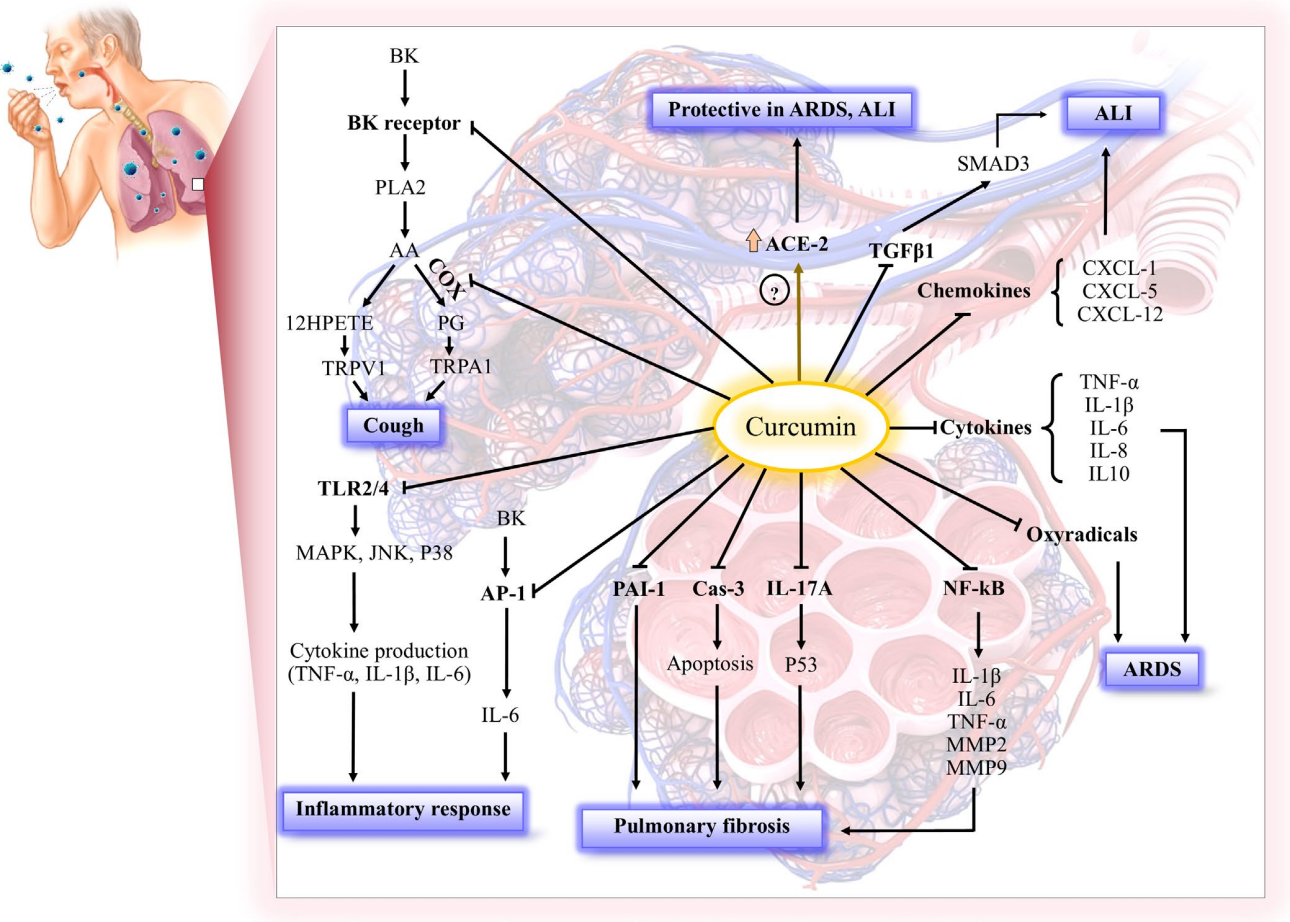
Possible molecular mechanisms of curcumin against COVID‐19 in the pulmonary system. AA: arachidonic acid, ALI: acute lung injury, AP‐1: activator protein 1, BK: bradykinin, ACE2: angiotensin‐converting enzyme 2, Ang II: angiotensin II, ARDS: acute respiratory distress syndrome, Cas‐3: caspase 3, COX: cyclooxygenase, CXCL: chemokine (C–X–C motif) ligand, 12‐HPETE: 12‐hydroperoxyeicosatetraenoic acid, JNK: c‐Jun N‐terminal kinase, 12 LOX: 12‐lipoxygenase, MMP: matrix metalloproteinase NF‐κB: nuclear factor kappa‐light‐chain‐enhancer of activated B cells, MAPK: mitogen‐activated protein kinase, PAI‐1: plasminogen activator inhibitor‐1, PLA2: phospholipase A2, PG: prostaglandin, SMAD3: mothers against decapentaplegic homolog 3, TGF‐β1: transforming growth factor‐beta 1, TNF‐α: tumor necrosis factor‐α, TLR: Toll‐like receptor, TRPA1: transient receptor potential channel subfamily vanilloid member 1, TRPV1: transient receptor potential channel subfamily A member 1

In this section, we summarize the important molecular mechanisms of curcumin that show potential ability against COVID‐19. Figure 3 presents a summary of the possible molecular mechanisms of curcumin against COVID‐19 via different signaling pathways in the pulmonary system. This figure shows the inhibitory effects of curcumin on TLRs, NF‐κB, cytokines, chemokines, bradykinin, oxyradicals, transforming growth factor‐beta1 (TGF‐β1), cyclooxygenase (COX), plasminogen activator inhibitor‐1 (PAI‐1), IL‐17A, and caspase‐3 (Cas‐3).

### 5.6 Antioxidant effects

In severe COVID‐19 infection, pneumonia may cause hypoxemia, which, in turn, disturbs cell metabolism and reduces the energy supply, and increases anaerobic fermentation. Then, acidosis happens and causes oxygen free radicals to destroy the phospholipid layer of the cell membrane (Li, Yang, et al., 2020). Therefore, treatment with a drug that has antioxidant properties will be good for these patients and curcumin has this effect. Several studies have shown that curcumin is a strong antioxidant (Abrahams, Haylett, Johnson, Carr, & Bardien, 2019; Farzaei et al., 2018; Mary, Vijayakumar, & Shankar, 2018; Trujillo et al., 2013). Curcumin (1 mg/kg, 5 mg/kg) increased the superoxide dismutase (SOD) level in acute lung injury induced by intestinal ischemia–reperfusion in mice (Fan et al., 2015). Furthermore, curcumin (200 mg/kg) reduced malondialdehyde (MDA) level and recovered the levels of xanthine oxidase (XO) and total antioxidative capacity (TAOC) in ventilator‐induced lung injury in rats (Wang, An, et al., 2018). Similarly, curcumin (200 mg/kg) increased SOD activity and decreased MDA content in the lung in acute injury induced by sepsis (Xiao, Yang, Sun, & Sun, 2012).

### 5.7 The anti‐inflammatory effects in acute lung injury/acute respiratory distress syndrome (ALI/ARDS) models

ARDS is a clinical syndrome and is associated with increased permeability pulmonary edema, severe arterial hypoxemia, and impaired carbon dioxide excretion, eventually resulting in respiratory failure. It may occur due to a pulmonary or extrapulmonary infectious or noninfectious insult (Matthay, Ware, & Zimmerman, 2012).

Major inflammatory mediators in ADRS include cytokines (TNF‐α, IL‐1β, IL‐6, IL‐8, IL‐10), chemokines such as macrophage inhibitory factor (MIF), and macrophage chemoattractant protein, metabolites of arachidonic acid (prostanoids and leukotrienes), and oxyradicals. Until yet, mechanical ventilation is only a proven strategy for treatment to improve the survival of patients (Matuschak & Lechner, 2010). Recently, rescue therapy with high‐dose vitamin C has been suggested to use for these patients (Fowler et al., 2019). Therefore, finding new treatments to overcome these mediators and preventing respiratory failure are necessary. On the other hand, the protective effects of curcumin were studied in several pulmonary diseases such as COPD, ARDS, pulmonary fibrosis, and asthma in animal studies (Lelli, Sahebkar, Johnston, & Pedone, 2017; Venkatesan, Punithavathi, & Babu, 2007). In this part of the review, we explain molecular mechanisms that curcumin may be useful to prevent or treat the ARDS.

#### 5.7.1 NF‐κB activity and inflammatory cytokines and chemokines

ALI is a model that is used for the ARDS animal study (Karunaweera, Raju, Gyengesi, & Münch, 2015; Olivera et al., 2012; Tian et al., 2006; Wang, Tang, Duan, & Yang, 2018), and curcumin exhibits its effects by predominantly targeting proinflammatory NF‐ĸB pathway (Ahn, Sethi, Jain, Jaiswal, & Aggarwal, 2006; Karunaweera et al., 2015; Olivera et al., 2012; Puar et al., 2018; Wang, Tang, et al., 2018). Curcumin decreased IL‐6 level and myeloperoxidase (MPO) activity, intercellular adhesion molecule‐1 (ICAM‐1) expression, and bronchoalveolar lavage fluid (BALF) protein in ALI induced by intestinal ischemia–reperfusion in mice that all of them are known as inflammatory indexes. It seems that curcumin by inhibiting NF‐κB could have anti‐inflammatory effects (Fan et al., 2015). Curcumin not only decreased the level of keratinocyte‐derived chemokine (KC), IL‐1β, macrophage inflammatory protein (MIP)‐2, TNF‐α, IL‐6, and TGF‐β in the BALF but also downregulated the expression of their genes except IL‐6 in ALI induced by *Staphylococcus aureus* in mice. Also, curcumin inhibited the activation of NF‐κB by downregulating phosphorylation of IκB‐α in bone marrow‐derived macrophages (BMDM) that were stimulated with *S. aureus*. It has been suggested that some part of the anti‐inflammatory effects of curcumin are due to regulating NF‐κB activity (Xu et al., 2015).

Curcumin downregulated the production of proinflammatory cytokine (TNF‐α, IFN‐α, and IL‐6) in influenza A virus‐infected human macrophages and BAL fluid of infected mice. Similar to other noted studies, curcumin downregulated the expression of NF‐κB and increased the cytosolic IκBα and inhibited its phosphorylation in the cytoplasm in human macrophages (Xu & Liu, 2017). In this way, curcumin reduced TNF‐α, MIP‐2, and IL‐6 in lipopolysaccharide (LPS)‐induced ALI in mice. It has been suggested that curcumin inhibits the release of cytokines by activation of 5′ adenosine monophosphate (AMP)‐activated protein kinase (AMPK) (Kim et al., 2016).

Curcumin reduced the production of TNF‐α, IL‐1β, IL‐6, and IL‐8, MMP‐2, and MMP‐9 both in mice and in A549 cells infected with influenza A virus. These cytokines exacerbate the ALI (Dai et al., 2018). The inhibitory role of curcumin on the expression of proinflammatory cytokines such as TNF‐α, IL‐1, and IL‐6 was reviewed in ALI and fibrosis by Gouda and Bhandary (2019). It seems that the most important molecular mechanism of curcumin on IL‐6 activities may be related to the downregulation or inhibition of IL‐6 signaling in different inflammatory diseases (Ghandadi & Sahebkar, 2017). Furthermore, curcumin has an inhibitory effect on IL‐17 A that plays a pivotal role in the inflammation of the alveolar epithelial cells in ALI studies. In other words, IL‐17 by activating P53 causes the stabilization of the PAI‐1, which in turn mediates the accumulation of extracellular matrix (ECM) and subsequent development of pulmonary fibrosis in alveolar type II (ATII) cells, and curcumin inhibits IL‐17A‐mediated changes in the p53‐fibrinolytic system (Figure 3) (Gouda & Bhandary, 2018, 2019). Curcumin also reduced the gene expression of chemokines such as chemokine (C‐X‐C motif) ligand 1 (CXCL1), CXCL5, and CXCL12 that is increased during inflammation in the airway epithelial cells in bleomycin‐induced ALI in mice (refer to Figure 3 for more details) (Gouda & Bhandary, 2018).

#### 5.7.2 TLRs in lung injury

The inhibitory effects of curcumin on the different subtypes of TLRs including extracellular TLR2 and TLR4 and TLR8 and intracellular TLR9 have been reported, which result in the therapeutic effects of curcumin in inflammation, infection, autoimmune, and ischemic disease (Boozari, Butler, & Sahebkar, 2019). Curcumin at low concentrations (10, 20 µM) prevented apoptosis and cytokine production (TNF‐α, IL‐6) induced by 19‑kDa *Mycobacterium tuberculosis* protein (P19) in human macrophages. Curcumin also reduced the expression of TLR2/JNK that may be involved in the apoptosis of macrophages (Li et al., 2014). ALI/ARDS could be the consequence of severe influenza A virus infection with substantial morbidity and mortality. On the other hand, curcumin decreased TLR2/4 gene expression and inhibited phosphorylation of p38, JNK, and NF‐κB in infected A549 cells with influenza A virus. It seems that curcumin regulates the TLR‐MAPK/NF‐κB signaling pathways involved in the replication and influenza pneumonia (Figure 3). However, other mechanisms have been suggested for the antiviral effects of curcumin. Furthermore, curcumin increased the survival rate in infected mice with this virus (Dai et al., 2018).

#### 5.7.3 Antiapoptotic and antifibrotic effect

Pulmonary pathology of COVID‐19 pneumonia in two patients with lung cancer showed the edema and prominent proteinaceous exudates, vascular congestion, and inflammatory clusters with fibrinoid material. Also, reactive alveolar epithelial hyperplasia and fibroblastic proliferation were reported in them (Tian et al., 2020). On the other hand, PAI‐1 plays a key mediator role in pulmonary fibrosis. Furthermore, PAI‐1 and apoptosis have an important role in the progression and pathogenesis of pulmonary fibrosis (Johnson, Shaikh, Muneesa, Rashmi, & Bhandary, 2020). Therefore, this issue led us to point out the antiapoptotic and the antifibrotic effects of curcumin here. The antiapoptotic effects of curcumin were found in different organ injuries including diabetes, nephrotoxicity, intestinal inflammation, neurotoxicity with several mechanisms (Loganes et al., 2017; Qihui, Shuntian, Xin, Xiaoxia, & Zhongpei, 2020; Soetikno et al., 2019; Sun et al., 2014). Both antiapoptotic and antifibrotic effects of curcumin were shown on the ALI model in mice. Curcumin reduced the expression of p53, PAI‐1, and chemokines in bleomycin‐induced ALI. Also, curcumin inhibited apoptosis mediated by IL‐17 and downregulated cleaved caspase‐3 in alveolar epithelial cells. The results suggest that the cross talk between the inflammatory, fibrinolytic, and apoptotic pathways is interrupted by curcumin (Gouda & Bhandary, 2018). Similar results of curcumin were found with the bleomycin model in human alveolar basal epithelial cells (A549) (Gouda, Prabhu, & Bhandary, 2018). Also, curcumin decreased PAI‐1 with cytokines and chemokines in ALI induced by staphylococcus aureus (Xu et al., 2015). On the other hand, curcumin inhibited the expression of TGF‐β1 and SMAD3 pathway in ALI induced by sepsis in rats that may involve in the pathogenesis of ALI (Xu et al., 2013). Curcumin reduced expression fibrosis markers including smooth muscle actin (α‐SMA), and Tenascin‐C in reovirus 1/L‐induced ALI/ARDS in mice (Avasarala et al., 2013). Intranasal curcumin reduced matrix metalloproteinases‐9 (MMP‐9)/tissue inhibitors of metalloproteinase (TIMP‐1) expression and increased α‐SMA, as a myofibroblast marker, which involves in muscle thickening in paraquat lung injury model in mice. It seems that pretreatment of curcumin prevented the early phase of pulmonary fibrosis by inhibiting inflammatory cells and producing fibrotic factors (Tyagi, Dash, & Singh, 2016) (Figure 3).

### 5.8 The inhibitory effects of curcumin on bradykinin to suppress cough

Bradykinin has an important role in the inflammatory events during acute and chronic inflammatory diseases such as respiratory tract infection and asthma (Broadley, Blair, Kidd, Bugert, & Ford, 2010; Hewitt et al., 2016). Furthermore, it seems that bradykinin could trigger cough in these inflammatory diseases or other conditions such as in patients with cough associated with captopril and enalapril as ACE inhibitors (Hewitt et al., 2016; Katsumata, Sekizawa, Ujiie, Sasaki, & Takishima, 1991) Curcumin is an inhibitor of activated protein‐1 (AP‐1) (Singh & Aggarwal, 1995). Curcumin prevented the expression of IL‐6 induced by bradykinin in human airway smooth muscle cells via this inhibition (Huang, Tliba, Panettieri, & Amrani, 2003).

On the other hand, it has shown that curcumin has a greater affinity to bradykinin B_1_ receptor (BK1) with strong inhibition activity (Ki value = 2.173 µg/ml) compared with BK2 receptor (Ki value = 58 µg/ml) (Yimam et al., 2016). The possible molecular mechanism of bradykinin for sensitizing cough reflex is through activation B_2_ receptors, which in turn stimulate the release of COX and 12‐lipoxygenase (12‐LOX) metabolites; then, these metabolites activate transient receptor potential (TRP) channel subfamily vanilloid member 1 (TRPV1) and subfamily A member 1 (TRPA1) channels result in an increase in both cough response and airway obstruction (Al‐Shamlan & El‐Hashim, 2019) (Figure 3). On the other hand, there are many studies due to the inhibitory effects of curcumin on 5‐LOX and COX‐2 (Rao, 2007). Furthermore, curcumin has shown these inhibitory effects in the airway studies (Kumari, Singh, Dash, & Singh, 2019; Yuan, Liu, Ma, Zhang, & Xie, 2018). Thus, curcumin is likely to inhibit the activity of bradykinin by inhibiting the COX enzyme (Figure 3).

### 5.9 Bronchodilator effect of curcumin

Curcumin (20 mg/kg, p.o.) significantly inhibits ovalbumin (OVA)‐induced airway constriction and airway hyperreactivity to histamine in sensitized guinea pigs (Ram, Das, & Ghosh, 2003). Also, curcumin (2.5 and 5 mg/kg, intranasal) significantly reduced bronchoconstriction in the mouse model of asthma (Subhashini et al., 2013).

Moreover, *C. longa* extract (1.5, 3 mg/ml) reduced tracheal contractile response to OVA and maximum response to methacholine in rats. It also decreased interstitial fibrosis (Shakeri, Roshan, & Boskabady, 2020). Standard therapy with capsule curcumin 500 mg BD daily for 30 days in patients of bronchial asthma significantly improved forced expiratory volume one second (FEV1) compared with standard therapy. However, the mean scores for cough, dyspnea, wheezing, chest tightness, and nocturnal symptoms were insignificant. Curcumin is recommended to use as an add‐on therapy for bronchial asthma (Abidi, Gupta, Agarwal, Bhalla, & Saluja, 2014).

### 5.10 Effect of curcumin on ACE2 expression

Dietary administration of curcumin (150 mg kg^‐1^ day^‐1^, gavage during Ang II infusion) decreased the protein level of the AT1 receptor and enhanced the expression of the AT2 receptor/ACE2 and results in the attenuation of myocardial fibrosis in a rat model of angiotensin II infusion (Pang et al., 2015). These data suggest that similar events happen in the lung tissues to prevent fibrosis. However, this hypothesis needs further studies (Figure 3). Possibly curcumin will be useful in combination therapy with angiotensin‐converting enzyme (ACE) inhibitors and AT1 antagonist (angiotensin II receptor blockers) to overcome fibrosis in COVID‐19 patients. On the other hand, recently, Monteil et al. have revealed the human recombinant soluble ACE2 (hrsACE2) could inhibit the growth of SARS‐CoV‐2 in Vero‐E6 cells, human capillary, and kidney organoids through preventing entry into host cells. However, they did not study lung organoids that are the major target organ for COVID‐19 (Monteil et al., 2020).

## 6 ADVANTAGE OF CURCUMIN OVER THE OTHER NATURAL AGENTS

Advantage of curcumin over other important natural agents with reported anti‐inflammatory activities such as zerumbone (Prasannan et al., 2012), thymoquinone (Siveen, Mustafa, et al., 2014), honokiol (Rajendran et al., 2012), escin (Tan et al., 2010), pinitol (Sethi, Ahn, Sung, & Aggarwal, 2008), and tocotrienols (Siveen, Ahn, et al., 2014) is that it has additional antiviral, antiemetic, antinociceptive, antifatigue, and bronchodilator effects that previously has been discussed in this review. Also, it has significant protective effects in the ARDS model in animal studies. These mentioned effects help us to conclude that curcumin has the potential to be effective against COVID‐19 infection.

## 7 SAFETY AND BIOAVAILABILITY OF CURCUMIN

To date, over 100 clinical trials have been completed with curcumin and safety, tolerability, and outcome have been reported in all of them (Kunnumakkara et al., 2017). An oral dose of 500 mg (two times a day, 30 days) was reported safe for curcumin (Soleimani et al., 2018). Curcumin up to 8,000 mg/day was safe, tolerable, and effective in humans, and higher doses were with toxicity (Kunnumakkara et al., 2019; Shanmugam et al., 2015). Curcumin has low bioavailability, but a lot of data from clinical trials showed the high efficacy of curcumin or turmeric against several diseases (Kunnumakkara et al., 2019). However, various strategies are used including analogs of curcumin and formulations such as adjuvants, nanoparticles, liposomes, micelles, and phospholipid complexes to improve its bioavailability (Kunnumakkara et al., 2017). Recently, it has been shown that the encapsulation of curcumin into specific nanocarrier could enhance its therapeutic efficacy (Moballegh Nasery et al., 2020).

## 8 CONCLUSION

COVID‐19 is spreading worldwide, leading a pandemic. There is no definitive treatment yet for this disease. In this review, we summarized clinical and molecular mechanisms that curcumin might be effective to treat COVID‐19. Research evidence suggests that curcumin will be useful to treat patients especially in ARDS cases with high mortality risk. Curcumin has several therapeutic effects including antiviral, antinociceptive, anti‐inflammatory, antipyretic, and antifatigue effects with several molecular mechanisms such as antioxidant, antiapoptotic, antifibrotic effects, and inhibitory effects on NF‐κB, inflammatory cytokines and chemokines, Toll‐like receptors, and bradykinin. The importance of this review is due to the fact that curcumin is a nutraceutical that could be a new treatment option to combat the COVID‐19 pandemic. Designing the best formulation with high efficacy and good bioavailability is necessary. Further clinical studies should focus on curcumin against COVID‐19 infection.

## CONFLICT OF INTEREST

The authors have no conflict of interest to declare.

## ETHICAL APPROVAL

The study did not involve any human or animal testing.

## References

[fsn31858-bib-0001] Abidi, A. , Gupta, S. , Agarwal, M. , Bhalla, H. L. , & Saluja, M. (2014). Evaluation of efficacy of curcumin as an add‐on therapy in patients of bronchial asthma. Journal of Clinical and Diagnostic Research, 8(8), Hc19‐24 10.7860/jcdr/2014/9273.4705 PMC4190737 25302215

[fsn31858-bib-0002] Abrahams, S. , Haylett, W. L. , Johnson, G. , Carr, J. A. , & Bardien, S. (2019). Antioxidant effects of curcumin in models of neurodegeneration, aging, oxidative and nitrosative stress: A review. Neuroscience, 406, 1–21. 10.1016/j.neuroscience.2019.02.020 30825584

[fsn31858-bib-0003] Ahn, K. S. , Sethi, G. , Jain, A. K. , Jaiswal, A. K. , & Aggarwal, B. B. (2006). Genetic deletion of NAD(P)H:Quinone oxidoreductase 1 abrogates activation of nuclear factor‐kappaB, IkappaBalpha kinase, c‐Jun N‐terminal kinase, Akt, p38, and p44/42 mitogen‐activated protein kinases and potentiates apoptosis. The Journal of Biological Chemistry, 281(29), 19798–19808. 10.1074/jbc.M601162200 16682409

[fsn31858-bib-0004] Akbar, M. U. , Rehman, K. , Zia, K. M. , Qadir, M. I. , Akash, M. S. H. , & Ibrahim, M. (2018). Critical review on curcumin as a therapeutic agent: From traditional herbal medicine to an ideal therapeutic agent. Critical Reviews in Eukaryotic Gene Expression, 28(1), 17–24. 10.1615/CritRevEukaryotGeneExpr.2018020088 29773013

[fsn31858-bib-0005] Alamdari, N. , O'Neal, P. , & Hasselgren, P. O. (2009). Curcumin and muscle wasting: A new role for an old drug? Nutrition, 25(2), 125–129. 10.1016/j.nut.2008.09.002 19028079 PMC3258441

[fsn31858-bib-0006] Al‐Shamlan, F. , & El‐Hashim, A. Z. (2019). Bradykinin sensitizes the cough reflex via a B(2) receptor dependent activation of TRPV1 and TRPA1 channels through metabolites of cyclooxygenase and 12‐lipoxygenase. Respiratory Research, 20(1), 110 10.1186/s12931-019-1060-8 31170972 PMC6551914

[fsn31858-bib-0007] Avasarala, S. , Zhang, F. , Liu, G. , Wang, R. , London, S. D. , & London, L. (2013). Curcumin modulates the inflammatory response and inhibits subsequent fibrosis in a mouse model of viral‐induced acute respiratory distress syndrome. PLoS One, 8(2), e57285 10.1371/journal.pone.0057285 23437361 PMC3577717

[fsn31858-bib-0008] Boozari, M. , Butler, A. E. , & Sahebkar, A. (2019). Impact of curcumin on toll‐like receptors. J Cell Physiology, 234(8), 12471–12482. 10.1002/jcp.28103 PMC13484202 30623441

[fsn31858-bib-0009] Broadley, K. J. , Blair, A. E. , Kidd, E. J. , Bugert, J. J. , & Ford, W. R. (2010). Bradykinin‐induced lung inflammation and bronchoconstriction: Role in parainfluenze‐3 virus‐induced inflammation and airway hyperreactivity. The Journal of Pharmacology and Experimental Therapeutics, 335(3), 681–692. 10.1124/jpet.110.171876 20847038

[fsn31858-bib-0010] Chan, J.‐W. , Yuan, S. , Kok, K.‐H. , To, K.‐W. , Chu, H. , Yang, J. , … Yuen, K.‐Y. (2020). A familial cluster of pneumonia associated with the 2019 novel coronavirus indicating person‐to‐person transmission: A study of a family cluster. Lancet, 395(10223), 514–523. 10.1016/s0140-6736(20)30154-9 31986261 PMC7159286

[fsn31858-bib-0011] Chen, N. , Zhou, M. , Dong, X. , Qu, J. , Gong, F. , Han, Y. , … Zhang, L. I. (2020). Epidemiological and clinical characteristics of 99 cases of 2019 novel coronavirus pneumonia in Wuhan, China: A descriptive study. Lancet, 395(10223), 507–513. 10.1016/s0140-6736(20)30211-7 32007143 PMC7135076

[fsn31858-bib-0012] Cheng, K. , Yang, A. , Hu, X. , Zhu, D. , & Liu, K. (2018). Curcumin attenuates pulmonary inflammation in lipopolysaccharide induced acute lung injury in neonatal rat model by activating peroxisome proliferator‐activated receptor γ (PPARγ) Pathway. Medical Science Monitor, 24, 1178–1184. 10.12659/MSM.908714 29480285 PMC5839073

[fsn31858-bib-0013] Cui, J. , Li, F. , & Shi, Z. L. (2019). Origin and evolution of pathogenic coronaviruses. Nature Review Microbiology, 17(3), 181–192. 10.1038/s41579-018-0118-9 30531947 PMC7097006

[fsn31858-bib-0014] Dai, J. , Gu, L. , Su, Y. , Wang, Q. , Zhao, Y. , Chen, X. , … Li, K. (2018). Inhibition of curcumin on influenza A virus infection and influenzal pneumonia via oxidative stress, TLR2/4, p38/JNK MAPK and NF‐κB pathways. International Immunopharmacology, 54, 177–187. 10.1016/j.intimp.2017.11.009 29153953

[fsn31858-bib-0015] Derosa, G. , Maffioli, P. , Simental‐Mendía, L. E. , Bo, S. , & Sahebkar, A. (2016). Effect of curcumin on circulating interleukin‐6 concentrations: A systematic review and meta‐analysis of randomized controlled trials. Pharmacological Research, 111, 394–404. 10.1016/j.phrs.2016.07.004 27392742

[fsn31858-bib-0016] Eke‐Okoro, U. J. , Raffa, R. B. , Pergolizzi, J. V. Jr , Breve, F. , & Taylor, R. Jr (2018). Curcumin in turmeric: Basic and clinical evidence for a potential role in analgesia. Journal of Clinical Pharmacy and Therapeutics, 43(4), 460–466. 10.1111/jcpt.12703 29722036

[fsn31858-bib-0017] Fan, Z. , Yao, J. , Li, Y. , Hu, X. , Shao, H. , & Tian, X. (2015). Anti‐inflammatory and antioxidant effects of curcumin on acute lung injury in a rodent model of intestinal ischemia reperfusion by inhibiting the pathway of NF‐ κB. International Journal of Clinical and Experimental Pathology, 8(4), 3451–3459. 26097529 PMC4466916

[fsn31858-bib-0018] Farzaei, M. , Zobeiri, M. , Parvizi, F. , El‐Senduny, F. , Marmouzi, I. , Coy‐Barrera, E. , … Abdollahi, M. (2018). Curcumin in liver diseases: A systematic review of the cellular mechanisms of oxidative stress and clinical perspective. Nutrients, 10(7), 855 10.3390/nu10070855 PMC6073929 29966389

[fsn31858-bib-0019] Ferreira, V. H. , Nazli, A. , Dizzell, S. E. , Mueller, K. , & Kaushic, C. (2015). The anti‐inflammatory activity of curcumin protects the genital mucosal epithelial barrier from disruption and blocks replication of HIV‐1 and HSV‐2. PLoS One, 10(4), e0124903 10.1371/journal.pone.0124903 25856395 PMC4391950

[fsn31858-bib-0020] Fowler, A. A. , Truwit, J. D. , Hite, R. D. , Morris, P. E. , DeWilde, C. , Priday, A. , … Halquist, M. (2019). Effect of vitamin C infusion on organ failure and biomarkers of inflammation and vascular injury in patients with sepsis and severe acute respiratory failure: The CITRIS‐ALI randomized clinical trial. JAMA, 322(13), 1261–1270. 10.1001/jama.2019.11825 31573637 PMC6777268

[fsn31858-bib-0021] Franceschi, F. , Feregalli, B. , Togni, S. , Cornelli, U. , Giacomelli, L. , Eggenhoffner, R. , & Belcaro, G. (2016). A novel phospholipid delivery system of curcumin (Meriva®) preserves muscular mass in healthy aging subjects. European Review for Medical and Pharmacological Sciences, 20(4), 762–766. 26957282

[fsn31858-bib-0022] Gao, J. , Tian, Z. , & Yang, X. (2020). Breakthrough: Chloroquine phosphate has shown apparent efficacy in treatment of COVID‐19 associated pneumonia in clinical studies. Bioscience Trends, 14(1), 72–73. 10.5582/bst.2020.01047 32074550

[fsn31858-bib-0023] Gautret, P. , Lagier, J.‐C. , Parola, P. , Hoang, V. T. , Meddeb, L. , Mailhe, M. , … Raoult, D. (2020). Hydroxychloroquine and azithromycin as a treatment of COVID‐19: Results of an open‐label non‐randomized clinical trial. International Journal of Antimicrobial Agents, 56(1), 105949 10.1016/j.ijantimicag.2020.105949 32205204 PMC7102549

[fsn31858-bib-0024] Ghandadi, M. , & Sahebkar, A. (2017). Curcumin: An effective inhibitor of interleukin‐6. Current Pharmaceutical Design, 23(6), 921–931. 10.2174/1381612822666161006151605 27719643

[fsn31858-bib-0025] Gouda, M. M. , & Bhandary, Y. P. (2018). Curcumin down‐regulates IL‐17A mediated p53‐fibrinolytic system in bleomycin induced acute lung injury in vivo. Journal of Cell Biochemistry, 119(9), 7285–7299. 10.1002/jcb.27026 29775223

[fsn31858-bib-0026] Gouda, M. M. , & Bhandary, Y. P. (2019). Acute lung injury: IL‐17A‐mediated inflammatory pathway and its regulation by curcumin. Inflammation, 42(4), 1160–1169. 10.1007/s10753-019-01010-4 31011925

[fsn31858-bib-0027] Gouda, M. M. , Prabhu, A. , & Bhandary, Y. P. (2018). Curcumin alleviates IL‐17A‐mediated p53‐PAI‐1 expression in bleomycin‐induced alveolar basal epithelial cells. Journal of Cell Biochemistry, 119(2), 2222–2230. 10.1002/jcb.26384 28902433

[fsn31858-bib-0028] Gurwitz, D. (2020). Angiotensin receptor blockers as tentative SARS‐CoV‐2 therapeutics. Drug Development Research, 81(5), 537–540. 10.1002/ddr.21656 32129518 PMC7228359

[fsn31858-bib-0029] Haider, S. , Naqvi, F. , Tabassum, S. , Saleem, S. , Batool, Z. , Sadir, S. , … Ahmad, S. (2013). Preventive effects of curcumin against drug‐ and starvation‐induced gastric erosions in rats. Scientia Pharmaceutica, 81(2), 549–558. 10.3797/scipharm.1207-17 23833720 PMC3700082

[fsn31858-bib-0030] Hamming, I. , Timens, W. , Bulthuis, M. L. , Lely, A. T. , Navis, G. , & van Goor, H. (2004). Tissue distribution of ACE2 protein, the functional receptor for SARS coronavirus. A first step in understanding SARS pathogenesis. The Journal of Pathology, 203(2), 631–637. 10.1002/path.1570 15141377 PMC7167720

[fsn31858-bib-0031] He, F. , Deng, Y. , & Li, W. (2020). Coronavirus disease 2019: What we know? Journal of Medical Virology, 92(7), 719–725. 10.1002/jmv.25766 32170865 PMC7228340

[fsn31858-bib-0032] He, Y. , Zhou, Y. , Liu, S. , Kou, Z. , Li, W. , Farzan, M. , & Jiang, S. (2004). Receptor‐binding domain of SARS‐CoV spike protein induces highly potent neutralizing antibodies: Implication for developing subunit vaccine. Biochemical and Biophysical Research Communications, 324(2), 773–781. 10.1016/j.bbrc.2004.09.106 15474494 PMC7092904

[fsn31858-bib-0033] Hewitt, M. M. , Adams, G. Jr , Mazzone, S. B. , Mori, N. , Yu, L. , & Canning, B. J. (2016). Pharmacology of bradykinin‐evoked coughing in guinea pigs. Journal of Pharmacology and Experimental Therapeutics, 357(3), 620–628. 10.1124/jpet.115.230383 27000801 PMC4885511

[fsn31858-bib-0034] Holshue, M. L. , DeBolt, C. , Lindquist, S. , Lofy, K. H. , Wiesman, J. , Bruce, H. , … Pillai, S. K. (2020). First case of 2019 novel coronavirus in the United States. The New England Journal of Medicine, 382(10), 929–936. 10.1056/NEJMoa2001191 32004427 PMC7092802

[fsn31858-bib-0035] Hosseini, A. , & Hosseinzadeh, H. (2018). Antidotal or protective effects of *Curcuma longa* (turmeric) and its active ingredient, curcumin, against natural and chemical toxicities: A review. Biomedicine and Pharmacotherapy, 99, 411–421. 10.1016/j.biopha.2018.01.072 29367110

[fsn31858-bib-0036] Huang, C. D. , Tliba, O. , Panettieri, R. A. Jr , & Amrani, Y. (2003). Bradykinin induces interleukin‐6 production in human airway smooth muscle cells: Modulation by Th2 cytokines and dexamethasone. American Journal of Respiratory Cell and Molecular Biology, 28(3), 330–338. 10.1165/rcmb.2002-0040OC 12594059

[fsn31858-bib-0037] Huang, C. , Wang, Y. , Li, X. , Ren, L. , Zhao, J. , Hu, Y. I. , … Cao, B. (2020). Clinical features of patients infected with 2019 novel coronavirus in Wuhan, China. Lancet, 395(10223), 497–506. 10.1016/s0140-6736(20)30183-5 31986264 PMC7159299

[fsn31858-bib-0038] Huang, W.‐C. , Chiu, W.‐C. , Chuang, H.‐L. , Tang, D.‐W. , Lee, Z.‐M. , Wei, L. I. , … Huang, C.‐C. (2015). Effect of curcumin supplementation on physiological fatigue and physical performance in mice. Nutrients, 7(2), 905–921. 10.3390/nu7020905 25647661 PMC4344567

[fsn31858-bib-0039] Hui, D. S. C. , & Zumla, A. (2019). Severe acute respiratory syndrome: Historical, epidemiologic, and clinical features. Infectious Disease Clinics of North America, 33(4), 869–889. 10.1016/j.idc.2019.07.001 31668196 PMC7127569

[fsn31858-bib-0040] Jia, H. (2016). Pulmonary angiotensin‐converting enzyme 2 (ACE2) and inflammatory lung disease. Shock, 46(3), 239–248. 10.1097/shk.0000000000000633 27082314

[fsn31858-bib-0041] Johnson, S. , Shaikh, S. B. , Muneesa, F. , Rashmi, B. , & Bhandary, Y. P. (2020). Radiation induced apoptosis and pulmonary fibrosis: Curcumin an effective intervention? International Journal of Radiation Biology, 96(6), 709–717. 10.1080/09553002.2020.1739773 32149561

[fsn31858-bib-0042] Kamali Aghdam, M. , Jafari, N. , & Eftekhari, K. (2020). Novel coronavirus in a 15‐day‐old neonate with clinical signs of sepsis, a case report. Infectious Diseases, 52(6), 427–429. 10.1080/23744235.2020.1747634 32233816 PMC7157949

[fsn31858-bib-0043] Karunaweera, N. , Raju, R. , Gyengesi, E. , & Münch, G. (2015). Plant polyphenols as inhibitors of NF‐κB induced cytokine production‐a potential anti‐inflammatory treatment for Alzheimer's disease? Frontiers in Molecular Neuroscience, 8, 24 10.3389/fnmol.2015.00024 26136655 PMC4468843

[fsn31858-bib-0044] Katsumata, U. , Sekizawa, K. , Ujiie, Y. , Sasaki, H. , & Takishima, T. (1991). Bradykinin‐induced cough reflex markedly increases in patients with cough associated with captopril and enalapril. The Tohoku Journal of Experimental Medicine, 164(2), 103–109. 10.1620/tjem.164.103 1721246

[fsn31858-bib-0045] Kim, J. , Jeong, S. W. , Quan, H. , Jeong, C. W. , Choi, J. I. , & Bae, H. B. (2016). Effect of curcumin (*Curcuma longa* extract) on LPS‐induced acute lung injury is mediated by the activation of AMPK. Journal of Anesthesia, 30(1), 100–108. 10.1007/s00540-015-2073-1 26335543

[fsn31858-bib-0046] Kuba, K. , Imai, Y. , Rao, S. , Gao, H. , Guo, F. , Guan, B. , … Penninger, J. M. (2005). A crucial role of angiotensin converting enzyme 2 (ACE2) in SARS coronavirus‐induced lung injury. Nature Medicine, 11(8), 875–879. 10.1038/nm1267 PMC7095783 16007097

[fsn31858-bib-0047] Kumari, A. , Singh, D. K. , Dash, D. , & Singh, R. (2019). Intranasal curcumin protects against LPS‐induced airway remodeling by modulating toll‐like receptor‐4 (TLR‐4) and matrixmetalloproteinase‐9 (MMP‐9) expression via affecting MAP kinases in mouse model. Inflammopharmacology, 27(4), 731–748. 10.1007/s10787-018-0544-3 30470954

[fsn31858-bib-0048] Kunnumakkara, A. B. , Bordoloi, D. , Padmavathi, G. , Monisha, J. , Roy, N. K. , Prasad, S. , & Aggarwal, B. B. (2017). Curcumin, the golden nutraceutical: Multitargeting for multiple chronic diseases. British Journal of Pharmacology, 174(11), 1325–1348. 10.1111/bph.13621 27638428 PMC5429333

[fsn31858-bib-0049] Kunnumakkara, A. B. , Harsha, C. , Banik, K. , Vikkurthi, R. , Sailo, B. L. , Bordoloi, D. , … Aggarwal, B. B. (2019). Is curcumin bioavailability a problem in humans: Lessons from clinical trials. Expert Opinion on Drug Metabolism & Toxicology, 15(9), 705–733. 10.1080/17425255.2019.1650914 31361978

[fsn31858-bib-0050] Lelli, D. , Sahebkar, A. , Johnston, T. P. , & Pedone, C. (2017). Curcumin use in pulmonary diseases: State of the art and future perspectives. Pharmacological Research, 115, 133–148. 10.1016/j.phrs.2016.11.017 27888157

[fsn31858-bib-0051] Li, B. O. , Yang, J. , Zhao, F. , Zhi, L. , Wang, X. , Liu, L. , … Zhao, Y. (2020). Prevalence and impact of cardiovascular metabolic diseases on COVID‐19 in China. Clinical Research in Cardiology, 109(5), 531–538. 10.1007/s00392-020-01626-9 32161990 PMC7087935

[fsn31858-bib-0052] Li, G. , & De Clercq, E. (2020). Therapeutic options for the 2019 novel coronavirus (2019‐nCoV). Nature Reviews Drug Discovery, 19(3), 149–150. 10.1038/d41573-020-00016-0 32127666

[fsn31858-bib-0053] Li, M. , Wu, Z. , Niu, W. , Wan, Y. , Zhang, L. , Shi, G. , & Xi, X. (2014). The protective effect of curcumin against the 19‐kDa Mycobacterium tuberculosis protein‐induced inflammation and apoptosis in human macrophages. Molecular Medicine Reports, 10(6), 3261–3267. 10.3892/mmr.2014.2615 25310360

[fsn31858-bib-0054] Li, Y. C. , Bai, W. Z. , & Hashikawa, T. (2020). The neuroinvasive potential of SARS‐CoV2 may play a role in the respiratory failure of COVID‐19 patients. Journal of Medical Virology, 92(6), 552–555. 10.1002/jmv.25728 32104915 PMC7228394

[fsn31858-bib-0055] Lin, C.‐J. , Chang, L. , Chu, H.‐W. , Lin, H.‐J. , Chang, P.‐C. , Wang, R. Y. L. , … Huang, C.‐C. (2019). High amplification of the antiviral activity of curcumin through transformation into carbon quantum dots. Small (Weinheim an Der Bergstrasse, Germany), 15(41), e1902641 10.1002/smll.201902641 31468672

[fsn31858-bib-0056] Liu, Z. , Huang, P. , Law, S. , Tian, H. , Leung, W. , & Xu, C. (2018). Preventive effect of curcumin against *chemotherapy*‐induced side‐effects. Frontiers in Pharmacology, 9, 1374 10.3389/fphar.2018.01374 30538634 PMC6277549

[fsn31858-bib-0057] Łoczechin, A. , Séron, K. , Barras, A. , Giovanelli, E. , Belouzard, S. , Chen, Y.‐T. , … Szunerits, S. (2019). Functional carbon quantum dots as medical countermeasures to human coronavirus. ACS Applied Materials & Interfaces, 11(46), 42964–42974. 10.1021/acsami.9b15032 31633330 PMC7075527

[fsn31858-bib-0058] Loganes, C. , Lega, S. , Bramuzzo, M. , Vecchi Brumatti, L. , Piscianz, E. , Valencic, E. , … Marcuzzi, A. (2017). Curcumin anti‐apoptotic action in a model of intestinal epithelial inflammatory damage. Nutrients, 9(6), 578 10.3390/nu9060578 PMC5490557 28587282

[fsn31858-bib-0059] Lu, R. , Zhao, X. , Li, J. , Niu, P. , Yang, B. O. , Wu, H. , … Tan, W. (2020). Genomic characterisation and epidemiology of 2019 novel coronavirus: Implications for virus origins and receptor binding. Lancet, 395(10224), 565–574. 10.1016/s0140-6736(20)30251-8 32007145 PMC7159086

[fsn31858-bib-0060] Mary, C. P. V. , Vijayakumar, S. , & Shankar, R. (2018). Metal chelating ability and antioxidant properties of curcumin‐metal complexes ‐ A DFT approach. Journal of Molecular Graphics & Modelling, 79, 1–14. 10.1016/j.jmgm.2017.10.022 29127853

[fsn31858-bib-0061] Matthay, M. A. , Ware, L. B. , & Zimmerman, G. A. (2012). The acute respiratory distress syndrome. The Journal of Clinical Investigation, 122(8), 2731–2740. 10.1172/jci60331 22850883 PMC3408735

[fsn31858-bib-0062] Matuschak, G. M. , & Lechner, A. J. (2010). Acute lung injury and the acute respiratory distress syndrome: Pathophysiology and treatment. Missouri Medicine, 107(4), 252–258. 20806836 PMC6188356

[fsn31858-bib-0063] Mo, P. , Xing, Y. , Xiao, Y. U. , Deng, L. , Zhao, Q. , Wang, H. , … Zhang, Y. (2020). Clinical characteristics of refractory COVID‐19 pneumonia in Wuhan, China. Clinical Infectious Diseases. 10.1093/cid/ciaa270 PMC7184444 32173725

[fsn31858-bib-0064] Moballegh Nasery, M. , Abadi, B. , Poormoghadam, D. , Zarrabi, A. , Keyhanvar, P. , Khanbabaei, H. , … Sethi, G. (2020). Curcumin delivery mediated by bio‐based nanoparticles: A review. Molecules, 25(3), 689 10.3390/molecules25030689 PMC7037405 32041140

[fsn31858-bib-0065] Moghadamtousi, S. Z. , Kadir, H. A. , Hassandarvish, P. , Tajik, H. , Abubakar, S. , & Zandi, K. (2014). A review on antibacterial, antiviral, and antifungal activity of curcumin. BioMed Research International, 2014, 186864 10.1155/2014/186864 24877064 PMC4022204

[fsn31858-bib-0066] Monteil, V. , Kwon, H. , Prado, P. , Hagelkrüys, A. , Wimmer, R. A. , Stahl, M. , … Penninger, J. M. (2020). Inhibition of SARS‐CoV‐2 infections in engineered human tissues using clinical‐grade soluble human ACE2. Cell, 81(4), 905–913. 10.1016/j.cell.2020.04.004 PMC7181998 32333836

[fsn31858-bib-0067] Morris, G. , Puri, B. K. , Walker, A. J. , Maes, M. , Carvalho, A. F. , Walder, K. , … Berk, M. (2019). Myalgic encephalomyelitis/chronic fatigue syndrome: From pathophysiological insights to novel therapeutic opportunities. Pharmacological Research, 148, 104450 10.1016/j.phrs.2019.104450 31509764

[fsn31858-bib-0068] Nassar, M. S. , Bakhrebah, M. A. , Meo, S. A. , Alsuabeyl, M. S. , & Zaher, W. A. (2018). Middle East respiratory syndrome coronavirus (MERS‐CoV) infection: Epidemiology, pathogenesis and clinical characteristics. European Review for Medical and Pharmacological Sciences, 22(15), 4956–4961. 10.26355/eurrev_201808_15635 30070331

[fsn31858-bib-0069] Nicol, L. M. , Rowlands, D. S. , Fazakerly, R. , & Kellett, J. (2015). Curcumin supplementation likely attenuates delayed onset muscle soreness (DOMS). European Journal of Applied Physiology, 115(8), 1769–1777. 10.1007/s00421-015-3152-6 25795285

[fsn31858-bib-0070] Olivera, A. , Moore, T. W. , Hu, F. , Brown, A. P. , Sun, A. , Liotta, D. C. , … Pace, T. W. W. (2012). Inhibition of the NF‐κB signaling pathway by the curcumin analog, 3,5‐Bis(2‐pyridinylmethylidene)‐4‐piperidone (EF31): Anti‐inflammatory and anti‐cancer properties. International Immunopharmacology, 12(2), 368–377. 10.1016/j.intimp.2011.12.009 22197802 PMC3372981

[fsn31858-bib-0071] Palizgir, M. T. , Akhtari, M. , Mahmoudi, M. , Mostafaei, S. , Rezaiemanesh, A. , & Shahram, F. (2018). Curcumin reduces the expression of interleukin 1β and the production of interleukin 6 and tumor necrosis factor alpha by M1 macrophages from patients with Behcet's disease. Immunopharmacology and Immunotoxicology, 40(4), 297–302. 10.1080/08923973.2018.1474921 29806793

[fsn31858-bib-0072] Pandaran Sudheeran, S. , Jacob, D. , Natinga Mulakal, J. , Gopinathan Nair, G. , Maliakel, A. , Maliakel, B. , … Im, K. (2016). Safety, tolerance, and enhanced efficacy of a bioavailable formulation of curcumin with fenugreek dietary fiber on occupational stress: A randomized, double‐blind, placebo‐controlled pilot study. Journal of Clinical Psychopharmacology, 36(3), 236–243. 10.1097/jcp.0000000000000508 27043120

[fsn31858-bib-0073] Pang, X. F. , Zhang, L. H. , Bai, F. , Wang, N. P. , Garner, R. E. , McKallip, R. J. , & Zhao, Z. Q. (2015). Attenuation of myocardial fibrosis with curcumin is mediated by modulating expression of angiotensin II AT1/AT2 receptors and ACE2 in rats. Drug Design, Development and Therapy, 9, 6043–6054. 10.2147/dddt.S95333 PMC4651552 26648693

[fsn31858-bib-0074] Praditya, D. , Kirchhoff, L. , Brüning, J. , Rachmawati, H. , Steinmann, J. , & Steinmann, E. (2019). Anti‐infective properties of the golden spice curcumin. Frontiers in Microbiology, 10, 912 10.3389/fmicb.2019.00912 31130924 PMC6509173

[fsn31858-bib-0075] Prasannan, R. , Kalesh, K. A. , Shanmugam, M. K. , Nachiyappan, A. , Ramachandran, L. , Nguyen, A. H. , … Sethi, G. (2012). Key cell signaling pathways modulated by zerumbone: Role in the prevention and treatment of cancer. Biochemical Pharmacology, 84(10), 1268–1276. 10.1016/j.bcp.2012.07.015 22842489

[fsn31858-bib-0076] Puar, Y. R. , Shanmugam, M. K. , Fan, L. , Arfuso, F. , Sethi, G. , & Tergaonkar, V. (2018). Evidence for the involvement of the master transcription factor NF‐κB in cancer initiation and progression. Biomedicines, 6(3), 82 10.3390/biomedicines6030082 PMC6163404 30060453

[fsn31858-bib-0077] Qian, G.‐Q. , Yang, N.‐B. , Ding, F. , Ma, A. H. Y. , Wang, Z.‐Y. , Shen, Y.‐F. , … Chen, X.‐M. (2020). Epidemiologic and clinical characteristics of 91 hospitalized patients with COVID‐19 in Zhejiang, China: A retrospective, multi‐centre case series. QJM, 113(7), 474–481. 10.1093/qjmed/hcaa089 32181807 PMC7184349

[fsn31858-bib-0078] Qihui, L. , Shuntian, D. , Xin, Z. , Xiaoxia, Y. , & Zhongpei, C. (2020). Protection of curcumin against streptozocin‐induced pancreatic cell destruction in T2D rats. Planta Medica, 86(2), 113–120. 10.1055/a-1046-1404 31801161

[fsn31858-bib-0079] Qin, C. , Zhou, L. , Hu, Z. , Zhang, S. , Yang, S. , Tao, Y. , … Tian, D. S. (2020). Dysregulation of immune response in patients with COVID‐19 in Wuhan. China. Clinical Infectious Diseases, 71(15), 762–768. 10.1093/cid/ciaa248 32161940 PMC7108125

[fsn31858-bib-0080] Qin, Y. , Lin, L. , Chen, Y. , Wu, S. , Si, X. , Wu, H. , … Zhong, Z. (2014). Curcumin inhibits the replication of enterovirus 71 in vitro. Acta Pharmaceutica Sinica B, 4(4), 284–294. 10.1016/j.apsb.2014.06.006 26579397 PMC4629085

[fsn31858-bib-0081] Rajendran, P. , Li, F. , Shanmugam, M. K. , Vali, S. , Abbasi, T. , Kapoor, S. , … Sethi, G. (2012). Honokiol inhibits signal transducer and activator of transcription‐3 signaling, proliferation, and survival of hepatocellular carcinoma cells via the protein tyrosine phosphatase SHP‐1. Journal of Cellular Physiology, 227(5), 2184–2195. 10.1002/jcp.22954 21792937

[fsn31858-bib-0082] Ram, A. , Das, M. , & Ghosh, B. (2003). Curcumin attenuates allergen‐induced airway hyperresponsiveness in sensitized guinea pigs. Biological and Pharmaceutical Bulletin, 26(7), 1021–1024. 10.1248/bpb.26.1021 12843631

[fsn31858-bib-0083] Rao, C. V. (2007). Regulation of COX and LOX by curcumin. Advances in Experimental Medicine and Biology, 595, 213–226. 10.1007/978-0-387-46401-5_9 17569213

[fsn31858-bib-0084] Richards, E. M. , & Raizada, M. K. (2018). ACE2 and pACE2: A pair of aces for pulmonary arterial hypertension treatment? American Journal of Respiratory and Critical Care Medicine, 198(4), 422–423. 10.1164/rccm.201803-0569ED 29634285 PMC6118027

[fsn31858-bib-0085] Rodriguez‐Morales, A. J. , Cardona‐Ospina, J. A. , Gutiérrez‐Ocampo, E. , Villamizar‐Peña, R. , Holguin‐Rivera, Y. , Escalera‐Antezana, J. P. , … Sah, R. (2020). Clinical, laboratory and imaging features of COVID‐19: A systematic review and meta‐analysis. Travel Medicine and Infectious Disease, 34, 101623 10.1016/j.tmaid.2020.101623 32179124 PMC7102608

[fsn31858-bib-0086] Sahebkar, A. , Cicero, A. F. G. , Simental‐Mendía, L. E. , Aggarwal, B. B. , & Gupta, S. C. (2016). Curcumin downregulates human tumor necrosis factor‐α levels: A systematic review and meta‐analysis of randomized controlled trials. Pharmacological Research, 107, 234–242. 10.1016/j.phrs.2016.03.026 27025786

[fsn31858-bib-0087] Sethi, G. , Ahn, K. S. , Sung, B. , & Aggarwal, B. B. (2008). Pinitol targets nuclear factor‐kappaB activation pathway leading to inhibition of gene products associated with proliferation, apoptosis, invasion, and angiogenesis. Molecular Cancer Therapeutics, 7(6), 1604–1614. 10.1158/1535-7163.MCT-07-2424 18566231

[fsn31858-bib-0088] Shakeri, F. , Roshan, N. M. , & Boskabady, M. H. (2020). Hydro‐ethanolic extract of *Curcuma longa* affects tracheal responsiveness and lung pathology in ovalbumin‐sensitized rats. International Journal for Vitamin and Nutrition Research, 90(1–2), 141–150. 10.1024/0300-9831/a000524 30789805

[fsn31858-bib-0089] Shanmugam, M. , Rane, G. , Kanchi, M. , Arfuso, F. , Chinnathambi, A. , Zayed, M. , … Sethi, G. (2015). The multifaceted role of curcumin in cancer prevention and treatment. Molecules, 20(2), 2728–2769. 10.3390/molecules20022728 25665066 PMC6272781

[fsn31858-bib-0090] Singh, S. , & Aggarwal, B. B. (1995). Activation of transcription factor NF‐kappa B is suppressed by curcumin (diferuloylmethane) [corrected]. The Journal of Biological Chemistry, 270(42), 24995–25000. 10.1074/jbc.270.42.24995 7559628

[fsn31858-bib-0091] Siveen, K. S. , Ahn, K. S. , Ong, T. H. , Shanmugam, M. K. , Li, F. , Yap, W. N. , … Sethi, G. (2014). Y‐tocotrienol inhibits angiogenesis‐dependent growth of human hepatocellular carcinoma through abrogation of AKT/mTOR pathway in an orthotopic mouse model. Oncotarget, 5(7), 1897–1911. 10.18632/oncotarget.1876 24722367 PMC4039111

[fsn31858-bib-0092] Siveen, K. S. , Mustafa, N. , Li, F. , Kannaiyan, R. , Ahn, K. S. , Kumar, A. P. , … Sethi, G. (2014). Thymoquinone overcomes chemoresistance and enhances the anticancer effects of bortezomib through abrogation of NF‐κB regulated gene products in multiple myeloma xenograft mouse model. Oncotarget, 5(3), 634–648. 10.18632/oncotarget.1596 24504138 PMC3996662

[fsn31858-bib-0093] Soetikno, V. , Sari, S. , Ul Maknun, L. , Sumbung, N. , Rahmi, D. , Pandhita, B. , … Estuningtyas, A. (2019). Pre‐treatment with curcumin ameliorates cisplatin‐induced kidney damage by suppressing kidney inflammation and apoptosis in rats. Drug Research, 69(2), 75–82. 10.1055/a-0641-5148 29945277

[fsn31858-bib-0094] Soleimani, V. , Sahebkar, A. , & Hosseinzadeh, H. (2018). Turmeric (*Curcuma longa*) and its major constituent (curcumin) as nontoxic and safe substances: Review. Phytotherapy Research, 32(6), 985–995. 10.1002/ptr.6054 29480523

[fsn31858-bib-0095] Song, Z. , Xu, Y. , Bao, L. , Zhang, L. , Yu, P. , Qu, Y. , … Qin, C. (2019). From SARS to MERS, thrusting coronaviruses into the spotlight. Viruses, 11(1), 59 10.3390/v11010059 PMC6357155 30646565

[fsn31858-bib-0096] Subhashini, X. , Chauhan, P. S. , Kumari, S. , Kumar, J. P. , Chawla, R. , Dash, D. , … Singh, R. (2013). Intranasal curcumin and its evaluation in murine model of asthma. International Immunopharmacology, 17(3), 733–743. 10.1016/j.intimp.2013.08.008 24021755

[fsn31858-bib-0097] Sun, P. , Lu, X. , Xu, C. , Sun, W. , & Pan, B. (2020). Understanding of COVID‐19 based on current evidence. Journal of Medical Virology, 92(6), 548–551. 10.1002/jmv.25722 32096567 PMC7228250

[fsn31858-bib-0098] Sun, Q. , Jia, N. , Wang, W. , Jin, H. , Xu, J. , & Hu, H. (2014). Activation of SIRT1 by curcumin blocks the neurotoxicity of amyloid‐β25‐35 in rat cortical neurons. Biochemical and Biophysical Research Communications, 448(1), 89–94. 10.1016/j.bbrc.2014.04.066 24755072

[fsn31858-bib-0099] Talebpour, M. , Hadadi, A. , Oraii, A. , & Ashraf, H. (2020). Rationale and design of a registry in a referral and educational medical center in Tehran, Iran: Sina Hospital Covid‐19 registry (SHCo‐19R). Advanced Journal of Emergency Medicine., 4(2s), e53 10.22114/ajem.v0i0.361

[fsn31858-bib-0100] Tan, S. M. , Li, F. , Rajendran, P. , Kumar, A. P. , Hui, K. M. , & Sethi, G. (2010). Identification of beta‐escin as a novel inhibitor of signal transducer and activator of transcription 3/Janus‐activated kinase 2 signaling pathway that suppresses proliferation and induces apoptosis in human hepatocellular carcinoma cells. The Journal of Pharmacology and Experimental Therapeutics, 334(1), 285–293. 10.1124/jpet.110.165498 20378717

[fsn31858-bib-0101] Tan, W. S. D. , Liao, W. , Zhou, S. , Mei, D. , & Wong, W. F. (2018). Targeting the renin‐angiotensin system as novel therapeutic strategy for pulmonary diseases. Current Opinion in Pharmacology, 40, 9–17. 10.1016/j.coph.2017.12.002 29288933

[fsn31858-bib-0102] Tian, S. , Hu, W. , Niu, L. , Liu, H. , Xu, H. , & Xiao, S. Y. (2020). Pulmonary pathology of early‐Phase 2019 novel coronavirus (COVID‐19) pneumonia in two patients with lung cancer. Journal of Thoracic Oncology, 15(5), 700–704. 10.1016/j.jtho.2020.02.010 32114094 PMC7128866

[fsn31858-bib-0103] Tian, X. F. , Yao, J. H. , Li, Y. H. , Zhang, X. S. , Feng, B. A. , Yang, C. M. , & Zheng, S. S. (2006). Effect of nuclear factor kappa B on intercellular adhesion molecule‐1 expression and neutrophil infiltration in lung injury induced by intestinal ischemia/reperfusion in rats. World Journal of Gastroenterology, 12(3), 388–392. 10.3748/wjg.v12.i3.388 16489637 PMC4066056

[fsn31858-bib-0104] Trujillo, J. , Chirino, Y. I. , Molina‐Jijón, E. , Andérica‐Romero, A. C. , Tapia, E. , & Pedraza‐Chaverrí, J. (2013). Renoprotective effect of the antioxidant curcumin: Recent findings. Redox Biology, 1(1), 448–456. 10.1016/j.redox.2013.09.003 24191240 PMC3814973

[fsn31858-bib-0105] Tyagi, N. , Dash, D. , & Singh, R. (2016). Curcumin inhibits paraquat induced lung inflammation and fibrosis by extracellular matrix modifications in mouse model. Inflammopharmacology, 24(6), 335–345. 10.1007/s10787-016-0286-z 27766504

[fsn31858-bib-0106] van den Brand, J. M. , Smits, S. L. , & Haagmans, B. L. (2015). Pathogenesis of Middle East respiratory syndrome coronavirus. The Journal of Pathology, 235(2), 175–184. 10.1002/path.4458 25294366 PMC7167882

[fsn31858-bib-0107] Venkatesan, N. , Punithavathi, D. , & Babu, M. (2007). Protection from acute and chronic lung diseases by curcumin. Advances in Experimental Medicine and Biology, 595, 379–405. 10.1007/978-0-387-46401-5_17 17569221

[fsn31858-bib-0108] Wan, S. , Xiang, Y. I. , Fang, W. , Zheng, Y. U. , Li, B. , Hu, Y. , … Yang, R. (2020). Clinical features and treatment of COVID‐19 patients in northeast Chongqing. Journal of Medical Virology, 92(7), 797–806. 10.1002/jmv.25783 32198776 PMC7228368

[fsn31858-bib-0109] Wan, Y. , Shang, J. , Graham, R. , Baric, R. S. , & Li, F. (2020). Receptor recognition by the novel coronavirus from Wuhan: An analysis based on decade‐long structural studies of SARS coronavirus. Journal of Virology, 94(7), 10.1128/jvi.00127-20 PMC7081895 31996437

[fsn31858-bib-0110] Wang, D. , Hu, B. O. , Hu, C. , Zhu, F. , Liu, X. , Zhang, J. , … Peng, Z. (2020). Clinical characteristics of 138 hospitalized patients with 2019 novel coronavirus‐infected pneumonia in Wuhan. China. JAMA, 323(11), 1061–1069. 10.1001/jama.2020.1585 32031570 PMC7042881

[fsn31858-bib-0111] Wang, X. , An, X. , Wang, X. , Bao, C. , Li, J. , Yang, D. , & Bai, C. (2018). Curcumin ameliorated ventilator‐induced lung injury in rats. Biomedicine and Pharmacotherapy, 98, 754–761. 10.1016/j.biopha.2017.12.100 29571243

[fsn31858-bib-0112] Wang, Y. , Tang, Q. , Duan, P. , & Yang, L. (2018). Curcumin as a therapeutic agent for blocking NF‐κB activation in ulcerative colitis. Immunopharmacology and Immunotoxicology, 40(6), 476–482. 10.1080/08923973.2018.1469145 30111198

[fsn31858-bib-0113] Wang, Z. , Yang, B. , Li, Q. , Wen, L. , & Zhang, R. (2020). Clinical features of 69 cases with coronavirus disease 2019 in Wuhan. China. Clinical Infectious Diseases, 71(15), 769–777. 10.1093/cid/ciaa272 32176772 PMC7184452

[fsn31858-bib-0114] Wen, C.‐C. , Kuo, Y.‐H. , Jan, J.‐T. , Liang, P.‐H. , Wang, S.‐Y. , Liu, H.‐G. , … Yang, N.‐S. (2007). Specific plant terpenoids and lignoids possess potent antiviral activities against severe acute respiratory syndrome coronavirus. Journal of Medicine Chemistry, 50(17), 4087–4095. 10.1021/jm070295s 17663539

[fsn31858-bib-0115] Wrapp, D. , Wang, N. , Corbett, K. S. , Goldsmith, J. A. , Hsieh, C.‐L. , Abiona, O. , … McLellan, J. S. (2020). Cryo‐EM structure of the 2019‐nCoV spike in the prefusion conformation. Science, 367(6483), 1260–1263. 10.1126/science.abb2507 32075877 PMC7164637

[fsn31858-bib-0116] Wu, F. , Zhao, S. U. , Yu, B. , Chen, Y.‐M. , Wang, W. , Song, Z.‐G. , … Zhang, Y.‐Z. (2020). A new coronavirus associated with human respiratory disease in China. Nature, 579(7798), 265–269. 10.1038/s41586-020-2008-3 32015508 PMC7094943

[fsn31858-bib-0117] Wu, Z. , & McGoogan, J. M. (2020). Characteristics of and important lessons from the coronavirus disease 2019 (COVID‐19) outbreak in China: Summary of a report of 72 314 cases from the Chinese center for disease control and prevention. JAMA, 323(13), 1239 10.1001/jama.2020.2648 32091533

[fsn31858-bib-0118] Xiao, X. , Yang, M. , Sun, D. , & Sun, S. (2012). Curcumin protects against sepsis‐induced acute lung injury in rats. Journal of Surgical Research, 176(1), e31–39 10.1016/j.jss.2011.11.1032 22520056

[fsn31858-bib-0119] Xu, F. , Diao, R. , Liu, J. , Kang, Y. , Wang, X. , & Shi, L. (2015). Curcumin attenuates staphylococcus aureus‐induced acute lung injury. The Clinical Respiratory Journal, 9(1), 87–97. 10.1111/crj.12113 24460792

[fsn31858-bib-0120] Xu, F. , Lin, S. H. , Yang, Y. Z. , Guo, R. , Cao, J. , & Liu, Q. (2013). The effect of curcumin on sepsis‐induced acute lung injury in a rat model through the inhibition of the TGF‐β1/SMAD3 pathway. International Journal of Immunopharmacology, 16(1), 1–6. 10.1016/j.intimp.2013.03.014 23541743

[fsn31858-bib-0121] Xu, Y. , & Liu, L. (2017). Curcumin alleviates macrophage activation and lung inflammation induced by influenza virus infection through inhibiting the NF‐κB signaling pathway. Influenza and Other Respiratory Viruses, 11(5), 457–463. 10.1111/irv.12459 28646616 PMC5596526

[fsn31858-bib-0122] Xu, Z. , Shi, L. , Wang, Y. , Zhang, J. , Huang, L. , Zhang, C. , … Wang, F.‐S. (2020). Pathological findings of COVID‐19 associated with acute respiratory distress syndrome. Lancet Respiratory Medicine, 8(4), 420–422. 10.1016/s2213-2600(20)30076-x 32085846 PMC7164771

[fsn31858-bib-0123] Yao, Q. , Ye, X. , Wang, L. , Gu, J. , Fu, T. , Wang, Y. , … Guo, Y. (2013). Protective effect of curcumin on chemotherapy‐induced intestinal dysfunction. International Journal of Clinical and Experimental Pathology, 6(11), 2342–2349. 24228095 PMC3816802

[fsn31858-bib-0124] Yimam, M. , Lee, Y.‐C. , Moore, B. , Jiao, P. , Hong, M. , Nam, J.‐B. , … Jia, Q. I. (2016). Analgesic and anti‐inflammatory effects of UP1304, a botanical composite containing standardized extracts of *Curcuma longa* and *Morus alba* . Journal of Integrative Medicine, 14(1), 60–68. 10.1016/s2095-4964(16)60231-5 26778230

[fsn31858-bib-0125] Yu, F. , Du, L. , Ojcius, D. M. , Pan, C. , & Jiang, S. (2020). Measures for diagnosing and treating infections by a novel coronavirus responsible for a pneumonia outbreak originating in Wuhan, China. Microbes and Infection, 22(2), 74–79. 10.1016/j.micinf.2020.01.003 32017984 PMC7102556

[fsn31858-bib-0126] Yuan, J. , Liu, R. , Ma, Y. , Zhang, Z. , & Xie, Z. (2018). Curcumin attenuates airway inflammation and airway remolding by inhibiting nf‐κb signaling and COX‐2 in cigarette smoke‐induced COPD mice. Inflammation, 41(5), 1804–1814. 10.1007/s10753-018-0823-6 29961171

[fsn31858-bib-0127] Zhang, H. , Penninger, J. M. , Li, Y. , Zhong, N. , & Slutsky, A. S. (2020). Angiotensin‐converting enzyme 2 (ACE2) as a SARS‐CoV‐2 receptor: Molecular mechanisms and potential therapeutic target. Intensive Care Medicine, 46(4), 586–590. 10.1007/s00134-020-05985-9 32125455 PMC7079879

[fsn31858-bib-0128] Zhou, P. , Yang, X.‐L. , Wang, X.‐G. , Hu, B. , Zhang, L. , Zhang, W. , … Shi, Z.‐L. (2020). A pneumonia outbreak associated with a new coronavirus of probable bat origin. Nature, 579(7798), 270–273. 10.1038/s41586-020-2012-7 32015507 PMC7095418

[fsn31858-bib-0129] Zhu, J.‐Y. , Yang, X. , Chen, Y. , Jiang, Y. E. , Wang, S.‐J. , Li, Y. , … Han, H.‐Y. (2017). Curcumin suppresses lung cancer stem cells via inhibiting Wnt/β‐catenin and sonic hedgehog pathways. Phytotherapy Research, 31(4), 680–688. 10.1002/ptr.5791 28198062

[fsn31858-bib-0130] Zhu, N. A. , Zhang, D. , Wang, W. , Li, X. , Yang, B. O. , Song, J. , … Tan, W. (2020). A novel coronavirus from patients with pneumonia in China, 2019. New England Journal of Medicine, 382(8), 727–733. 10.1056/NEJMoa2001017 31978945 PMC7092803

[fsn31858-bib-0131] Zumla, A. , Chan, J. F. , Azhar, E. I. , Hui, D. S. , & Yuen, K. Y. (2016). Coronaviruses ‐ drug discovery and therapeutic options. Nature Reviews. Drug Discovery, 15(5), 327–347. 10.1038/nrd.2015.37 26868298 PMC7097181

